# Association of polymorphisms within the Renin-Angiotensin System with metabolic syndrome in a cohort of Chilean subjects

**DOI:** 10.1590/2359-3997000000134

**Published:** 2016-02-11

**Authors:** Christian L. Herrera, Wilma Castillo, Patricia Estrada, Bárbara Mancilla, Gerardo Reyes, Nicolás Saavedra, Neftalí Guzmán, Pamela Serón, Fernando Lanas, Luis A. Salazar

**Affiliations:** 1 Center of Molecular Biology and Pharmacogenetics, Scientific and Technological Bioresource Nucleus Universidad de La Frontera Temuco Chile Center of Molecular Biology and Pharmacogenetics, Scientific and Technological Bioresource Nucleus, Universidad de La Frontera (BIOREN-UFRO), Temuco, Chile; 2 Departamento de Ciencias Preclínicas Faculty of Medicine Universidad de La Frontera Temuco Chile Departamento de Ciencias Preclínicas, Faculty of Medicine, Universidad de La Frontera, Temuco, Chile; 3 Faculty of Health Sciences Universidad Católica de Temuco Temuco Chile Faculty of Health Sciences, Universidad Católica de Temuco, Temuco, Chile; 4 Departamento de Medicina Interna Faculty of Medicine Universidad de La Frontera Temuco Chile Departamento de Medicina Interna, Faculty of Medicine, Universidad de La Frontera, Temuco, Chile

**Keywords:** Metabolic syndrome, gender, polymorphisms, renin-angiotensin system

## Abstract

**Objective:**

: Metabolic syndrome (MetS) is associated with hypertension, obesity and dyslipidemia. Thus, genetic variants related with these conditions may modulate its development. We evaluated the effect of polymorphisms in the renin-angiotensin system (RAS) on metabolic syndrome risk in a cohort of Chilean subjects.

**Subjects and methods:**

: A total of 152 subjects, 83 with MetS (51.2 ± 9.6 years) and 69 without MetS (49.5 ± 9.3 years) of both genders were included, according to the ATP III update criteria. The rs4340 Insertion/Deletion (I/D), rs699 (T>C) and rs5186 (A>C) of the ACE, AGT and AGTR1 genes, respectively, were genotyped.

**Results:**

: After adjusting for age and gender, we observed the DD genotype of rs4340 associated with MetS (p = 0.02). Specifically, the DD genotype was associated with MetS risk in women (OR = 4.62, 95%CI, 1.41 – 15.04; p < 0.01). In males, the AA genotype for rs5186 variant was associated with an increased risk for developing MetS when compared with women carrying the same genotype (OR = 3.2; 95%CI, 1.03 – 9.89; p = 0.04). In subjects without MetS, DD genotype was associated with increased waist circumference (p = 0.023) while subjects with MetS carrying the rs5186 TT genotype showed higher levels of HDL-cholesterol (p = 0.031).

**Conclusion:**

: The present study contributes data highlighting the role for RAS polymorphisms in predisposing to metabolic syndrome in Chilean subjects.

## INTRODUCTION

The Renin-Angiotensin System (RAS) is related to the regulation of the corporal compartment volume, arterial pressure levels and kidney blood flow. However, this system is complex, and its functions are widely distributed in different tissues of our body which are currently not fully understood.

RAS polymorphisms have been widely studied in diverse populations. Although contradictory information exists, many authors relate them to various pathological conditions, apparently modulating the development of diseases like hypertension, chronic kidney disease, myocardial infarction and diabetes complications (1,2).

In the last years, the study of tissue RAS regulation sustained that this system can be a fundamental participant in metabolic processes and could explain the origin and complications of some metabolic disorders, such as Metabolic Syndrome (MetS) (3). MetS is a condition in which obesity and insulin resistance play major roles for its development, and the presence of this syndrome has been associated with a 2-fold increase in cardiovascular disease risk, and 5-fold increase in diabetes mellitus risk (4). Clinical definition of MetS corresponds to a consequence of the interaction between factors like central obesity, hyperglycemia, dyslipidemia and hypertension; however, this doesn’t consider the influence of sex on the pathophysiology and clinical expression of this condition (5). In Chile, the National Health Survey 2009-2010 showed a high 35.3% prevalence (to define MetS, the ATPIII update criteria was used, considering waist cutoffs validated for Chile), being highest in men (41.6% *vs* 30.9%) and increasing with age. However, at 65 years old the prevalence is equal in both men and women (53.0% *vs* 50.7%) (6). In the United States a similar behavior was observed; however, a significant increase in prevalence also occurred among younger women (7).

Reports about the relationship between RAS polymorphisms, particularly the rs4340, and MetS are few, and in some cases contradictory. As an example, in Chinese population Thomas and cols. pointed out an association between Insertion (I) allele with metabolic syndrome (8). On the other hand, Lee and Tsai*, *who studied diabetic individuals with and without MetS associated the Deletion (D) allele to MetS in patients with type 2 diabetes mellitus (DM2), and specifically with dyslipidemia, albuminuria and higher serum triglycerides levels (9). Complementarily, Yang and cols.*, *also in Chinese population demonstrated that interactions among RAS-related genes – particularly between *AGT* and *ACE* – were associated with DM2 (10). Reports from other populations are not so different. In Mexican population an association between DD genotype with MetS was identified, opposite to Polish population who do not show associations with insulin resistance, intensity of MetS or HOMA rate (11,12).

Another relevant polymorphism in the MetS context is rs699 (M235T). This variant was described by Procopciuc and cols. as a conditioning factor for MetS due to the combination of the CC genotype (TT235) with DD of *ACE*, or CC+AC rs5186 (A1166C) genotypes increase the risk for developing MetS (13).

One of the most studied RAS-related polymorphisms is the A1166C variant (rs5186). This is a SNP located in the 3-untranslated region (3-UTR) of the angiotensin II receptor type 1 gene (*AGTR1*), which is also known as AT2R1 or AT1R, corresponding to adenine (A) to cytosine (C) transversion at position 1166 (14). At present, abundant data extensively implicates the *AGTR1 *rs5186 SNP as a risk factor for hypertension (14-18).

As aforementioned, the influence of rs4340 (I/D *ACE*), rs699 and rs5186 polymorphisms in MetS remain unclear, and currently in Chile, the study of these variants is limited, existing few published articles which only evaluate the rs4340 variant and its relation with hypertension, cardiac structure and plasma neutral endopeptidase activity (19-21). Since Chilean population carries a different ethnical background, incorporating diverse proportions of Amerindian and European ancestors depending on the geographical origin of the studied population, genetic profiles can fluctuate and could explain patterns of morbidity (22). Therefore, we hypothesized that polymorphisms in candidate genes involved in the RAS system may contribute to MetS. So, we aimed to evaluate the effect of candidate gene polymorphisms of the RAS system -rs4043, rs699 and rs5186- on metabolic syndrome risk in a cohort of Chilean subjects.

## SUBJECTS AND METHODS

### Subjects

A sample of 152 individuals, both sexes, unrelated, aged 35-70 years, with a mean age of 50.5 ± 9.4 years, from La Araucanía region in Chile, were included in a cross-sectional study. All participants answered a questionnaire to get the family history of cardiovascular disease (CVD), morbid personal history and drugs consumption, among other data. Blood pressure, waist circumference, weight and height were also determined. In addition, a blood sample was obtained by venipuncture for biochemical determinations of glucose, cholesterol, triglycerides concentration and molecular analysis. The study protocol was approved by the Ethics Committee of the Araucanía Sur Health Service, and all participants gave written informed consent.

The sample of patients of both genders were distributed in two groups based in the presence (n = 83) or absence (n = 69) of metabolic syndrome was studied. The comparative analysis between individuals with and without metabolic syndrome (MetS), its association with polymorphisms studied and the effect of MetS components was considered according to the ATPIII update criteria, with waist cutoffs validated for Chile (6).

### Biochemical and molecular determinations

Blood samples were obtained after 12 hours of fasting. Serum was analyzed to determinate the level of total cholesterol, triglyceride and glucose using enzymatic colorimetric methods. HDL-cholesterol concentration was determined after precipitation of LDL and VLDL with phosphotungstic acid and magnesium using the CHOD-PAP method. LDL cholesterol concentration was calculated using the Friedewald formula. All determinations were obtained using a semiautomatic photometer (Humalyzer 3000, Wiesbaden, Germany).

Genomic DNA was extracted from peripheral blood leucocytes using a method previously described by Salazar and cols. (23). Integrity of genomic DNA was visualized by electrophoresis in 1.0% agarose gel. Three polymorphisms in genes of RAS were studied: rs5186 in angiotensin II type 1 receptor (*AGTR1*), rs699 in angiotensinogen (*AGT*) and rs4340 of angiotensin converter enzyme (*ACE*). The rs4340 polymorphism was determined using specific primers to detect insertion or deletion of nucleotide sequence (Forward 5’-CTG GAG AGC CAC TCC CAT CCT TTC T- 3’, Reverse 5’-GAC GTG GCC ATC ACA TTC GTC AGA T- 3’, annealing temperature: 64ºC). PCR products size were 490 bp and 190 bp for I and D allele respectively, then a single band of 490 or 190 bp confirmed a homozygous II and DD genotype respectively, and two bands of 490 and 190 bp confirmed heterozygous ID genotype. The gene polymorphism rs5186 was detected by Polymerase Chain Reaction following enzymatic restriction (PCR-RFLP). Primers used were forward 5’- ATA ATG TAA GCT CAT CCA CC- 3’ and reverse 5’- GAG ATT GCA TTT CTG TCA GT - 3’, annealing temperature: 56ºC. Using the restriction enzyme HpyF3I (*Dde*I, 5 Units, Thermo Scientific) the DNA fragments obtained for the genotypes were: AA: 350 bp; CC: 211 + 139 bp and AC: 350 + 211 + 139 bp. The rs699 polymorphism was detected by Polymerase Chain Reaction and enzymatic restriction (PCR-RFLP), the primers used were forward 5’- TGG ATG CGC ACA AGG TCC TGT- 3’, reverse 5’- CAG GGT GCT GTC CAC ACT GGC TCG C- 3’, annealing temperature: 64ºC. Using the restriction enzyme *Bsh*1236I (5 Units, Thermo Scientific) genotypes were: CC: 24 + 281 bp; CT: 24 + 281 + 305 bp and TT: 305 bp.

The PCR products and digestion fragments were identified by electrophoresis in agarose gel, stained with ethidium bromide (0.5 mg/L) and visualized on a UV transilluminator (E-Box 1000, Vilber Lourmat, France). In addition, all gels were reread blindly by two persons without any change, and 20% of the analyses were randomly repeated.

Management of biological samples and chemical reagents was performed following biosafety guidelines described in the Manual of Standards Biosafety of the National Commission of Scientific and Technological Research (CONICYT, Chile).

### Statistical analysis

To analyze the data we used the GraphPad Software for Windows, v. 5.0 (La Jolla, CA, USA). Data are presented as mean ± SD. Differences between the means of continuous variables were evaluated by Student *t*-test or ANOVA. The p-values were adjusted for co-variables (age and gender) using logistic regression analysis (SNPStats, Spain). The allelic frequencies and genotype distribution were estimated by gene counting. Differences between non continuous variables, genotype distribution, allele frequency, and Hardy-Weinberg equilibrium were tested by Fisher’s exact test or Chi-square test. Statistical significance was at *p* < 0.05.

## RESULTS

Details of clinical data and biochemical results are presented in Table 1. Significant higher values of blood pressure, weight, body mass index, obesity status, waist circumference, glycaemia and triglycerides, and lower levels of HDL-cholesterol were observed in MetS group. Subgroup analyses revealed only a significant difference in arterial blood pressure (Table 2).

**Table 1:** Clinical and biochemical characteristics of studied groups

	No MetS (n = 69)	MetS (n = 83)	p-value
Age, years	49.5 ± 9.3	51.2 ± 9.6	0.266
Men, n (%)	16 (24.6)	29 (34.9)	0.215
SBP (mmHg)	120.6 ± 18.8	136.4 ± 21.6	< 0.001
DBP (mmHg)	73.5 ± 9.4	83.0 ± 11.2	< 0.001
Height (cm)	157.9 ± 6.9	159.8 ± 8.9	0.140
Weight (kg)	69.6 ± 9.7	80.5 ± 15.5	< 0.001
BMI (kg/m^2^)	27.9 ± 3.4	31.5 ± 5.1	< 0.001
Normal Weight, n (%)	10 (14.5)	3 (3.6)	< 0.001
Overweight, n (%)	44 (63.8)	36 (43.4)	< 0.001
Obese, n (%)	15 (21.7)	44 (53.0)	< 0.001
Diabetes, n (%)	2 (2.9)	9 (10.8)	0.113
Waist Circumference (cm)	87.6 ± 9.4	98.5 ± 12.6	< 0.001
Glycaemia (mg/dL)	87.6 ± 8.7	104.5 ± 33.6	< 0.001
Total cholesterol (mg/dL)	206.4 ± 33.7	212.5 ± 44.4	0.476
HDL-cholesterol (mg/dL)	45.4 ± 9.1	37.1 ± 7.1	< 0.001
LDL-cholesterol (mg/dL)	138.7 ± 32.1	137.6 ± 42.4	0.749
Triglycerides (mg/dL)	111.3 ± 37.6	180.2 ± 83.1	< 0.001

MetS: metabolic syndrome; No MetS: no metabolic syndrome; SBP: systolic blood pressure; DBP: diastolic blood pressure; BMI: body mass index; HDL: high density lipoprotein; LDL: low density lipoprotein.

**Table 2:** Comparison of components of metabolic syndrome between subgroups of hypertensive and non-hypertensive subjects

	No MetS			MetS		
	
	HBP (n = 14)	No HBP (n = 55)	p-value	HBP (n = 39)	No HBP (n = 44)	p-value
SBP (mmHg)	140.7 ± 24.1	115.5 ± 13.1	**< 0.001**	150.7 ± 21.2	123.6 ± 11.9	**< 0.001**
DBP (mmHg)	79.9 ± 13.5	71.8 ± 7.4	**0.004**	88.6 ± 11.6	78.0 ± 8.1	**< 0.001**
Waist circumference (cm)	87.2 ± 9.5	87.7 ± 9.5	0.870	98.7 ± 12.0	98.3 ± 13.2	0.901
Glycaemia (mg/dL)	89.5 ± 9.1	87.2 ± 8.6	0.368	105.9 ± 31.5	103.2 ± 35.6	0.710
HDL-cholesterol (mg/dL)	49.1 ± 7.8	44.5 ± 9.3	0.091	37.4 ± 6.8	36.9 ± 7.5	0.795
Triglycerides (mg/dL)	120.4 ± 21.8	109.0 ± 40.5	0.315	172.1 ± 76.1	187.4 ± 89.2	0.404

HBP: high blood pressure; No HBP: no high blood pressure; MetS: metabolic syndrome; No MetS: no metabolic syndrome; SBP: systolic blood pressure; DBP: diastolic blood pressure.

Table 3 presents the genotypic and allelic frequencies for the *ACE*, *AGT* and *AGTR1* variants. Genotype distribution for the rs4340, rs699 and rs5186 polymorphisms did not differ between individuals with and without MetS in a crude analysis, however, after adjusting by age and gender we observed the DD genotype associated with MetS. Additionally, differences were observed in genotype and allelic distribution between women with and without MetS for the rs4340 polymorphism (Table 4). In a gender comparison, we observed that men carriers of the rs5186 AA variant have a higher risk for MetS (Table 5). The interaction between genotypes and its relation with MetS was evaluated, although no differences were detected.

**Table 3:** Distribution of RAS genotypes and alleles in Chilean subjects with and without metabolic syndrome (n = 152)

	No MetS	MetS	Odds ratio, CI (95%)	p-value	p-value Adjusted*
**rs4340**					
**Genotypes**					
II	27 (39.1)	32 (38.6)	1.03 (0.5-1.9)	1.00	0.91
ID	35 (50.7)	33 (39.8)	0.64 (0.3-1.2)	0.19	0.05
DD	7 (10.1)	18 (21.6)	2.45 (0.9-6.3)	0.08	**0.02**
**Alleles**					
I	89 (64.5)	97 (58.4)	1.29 (0.8-2.0)	0.29	
D	49 (35.5)	69 (41.6)			
					
**rs699**					
**Genotypes**					
TT	9 (13.4)	11 (13.3)	1.02 (0.4-2.6)	1.00	0.79
TC	32 (47.8)	43 (52.8)	1.18 (0.6-2.2)	0.74	0.86
CC	26 (38.8)	29 (35.9)	0.85 (0.4-1.7)	0.73	0.70
**Alleles**					
T	50 (37.3)	65 (39.2)	0.92 (0.6-1.5)	0.81	
C	84 (63.7)	101 (60.8)			
					
**rs5186**					
**Genotypes**					
AA	37 (54.4)	45 (54.2)	1.01 (0.5-1.9)	1.00	0.80
AC	28 (41.2)	33 (39.8)	0.94 (0.5-1.8)	0.86	0.76
CC	3 (4.4)	5 (6.0)	1.38 (0.3-6.0)	0.73	0.55
**Alleles**					
A	102 (75.0)	123 (74.1)	1.05 (0.6-1.8)	0.89	
C	34 (25.0)	43 (25.9)			

MetS: metabolic syndrome; No MetS: no metabolic syndrome. * *P*-value adjusted for sex and age.

**Table 4:** Distribution by gender of RAS genotypes and alleles in Chilean subjects with and without metabolic syndrome

Sex	Genotype	No MetS	MetS	Odds ratio, CI (95%)	p-value	p-value Adjusted*
	rs4340					
Women	II	23 (44.2)	20 (37.0)	1.35 (0.62-2.94)	0.55	0.33
	ID	25 (48.1)	19 (35.2)	0.59 (0.27-1.28)	0.24	**< 0.01**
	DD	4 (7.7)	15 (27.8)	4.62 (1.41-15.04)	**0.01**	**< 0.01**
						
	I	71 (68.3)	59 (54.6)	1.79 (1.02-3.13)	**0.048**	
	D	33 (31.7)	49 (45.4)			
Men	II	4 (23.5)	12 (41.4)	0.44 (0.11-1.67)	0.33	0.20
	ID	10 (58.8)	14 (48.3)	0.65 (0.19-2.19)	0.55	0.40
	DD	3 (17.6)	3 (16.3)	0.54 (0.09-3.03)	0.65	0.44
						
	I	18 (52.9)	38 (65.5)	0.59 (0.25-1.41)	0.27	
	D	16 (47.1)	20 (34.5)			
	rs699					
Women	TT	6 (11.8)	7 (13.0)	0.89 (0.28-2.87)	1.00	0.95
	CT	25 (49.0)	29 (53.7)	1.21 (0.56-2.59)	0.69	0.89
	CC	20 (39.2)	18 (33.3)	0.79 (0.35-1.72)	0.68	0.66
						
	T	37 (36.3)	43 (39.8)	0.86 (0.49-1.50)	0.67	
	C	65 (63.7)	65 (60.2)			
Men	TT	3 (18.8)	4 (13.8)	1.44 (0.27-7.44)	0.68	0.74
	CT	7 (43.8)	14 (48.3)	1.20 (0.35-4.09)	1.00	0.92
	CC	6 (37.5)	11 (37.9)	1.02 (0.29-3.59)	1.00	0.93
	T	13 (40.6)	22 (37.9)	1.12 (0.46-2.71)	0.82	
	C	19 (59.4)	36 (62.1)			
	rs5186					
Women	AA	32 (61.5)	30 (55.5)	1.28 (0.59-2.78)	0.55	0.56
	AC	18 (34.6)	19 (43.2)	1.03 (0.46-2.28)	1.00	0.48
	CC	2 (3.9)	5 (9.3)	2.55 (0.47-13.78)	0.43	0.23
	A	82 (78.8)	79 (73.1)	1.37 (0.73-2.58)	0.34	
	C	22 (21.2)	29 (26.9)			
Men	AA	5 (31.3)	15 (51.7)	0.42 (0.11-1.53)	0.22	0.22
	AC	10 (62.5)	14 (48.3)	0.56 (0.16-1.95)	0.53	0.20
	CC	1 (6.3)	0 (0.0)	NC	0.36	0.15
	A	20 (62.5)	44 (75.9)	0.53 (0.21-1.35)	0.20	
	C	12 (37.5)	14 (24.1)			

MetS: metabolic syndrome; No MetS: no metabolic syndrome. * *P*-value adjusted for age.

**Table 5:** Frequency of metabolic syndrome by gender and RAS-related genotypes

Gender		No MetS	MetS		No MetS	MetS		No MetS	MetS

	rs4340			rs699			rs5186		
Women	II	23	20	TT	6	7	AA	32	30
Men		4	12		3	4		5	15
		OR: 3.45 (0.96-12.4) p = 0.08			OR: 1.14 (0.17-7.28) p = 1,00			OR: 3.20 (1.03-9.89) **p = 0.04**	
Women	ID	25	19	TC	25	29	AC	18	19
Men		10	14		7	14		10	14
		OR: 1.84 (0.67-5.04) p = 0.31			OR: 1.72 (0.60-4.94) p = 0.44			OR: 1.3 (0.47-3.74) p = 0.61	
Women	DD	4	15	CC	20	18	CC	2	5
Men		3	3		6	11		1	0
		OR: 0.27 (0.04-1.86) p = 0.29			OR: 2.04 (0.63-6.64) p = 0.26			OR: 0.15 (0.00-5.18) p = 0.38	

OR: odds ratio; MetS: metabolic syndrome; No MetS: no metabolic syndrome.

The influence of genotypes on systolic blood pressure, diastolic blood pressure, waist circumference, glycaemia, HDL-cholesterol and triglycerides was evaluated; higher levels of waist circumference for DD genotype of rs4340 in individuals without MetS and in a similar way, a higher concentration of HDL-cholesterol for TT genotype of rs699 in individuals with MetS was observed (Table 6).

**Table 6:** Impact of rs4340, rs699 and rs5186 genotypes on the components of MetS in Chilean subjects

		MetS				No MetS		
**rs4340**	**II (n = 32)**	**ID (n = 33)**	**DD (n = 18)**	**p-value**	**II (n = 27)**	**ID (n = 35)**	**DD (n = 7)**	**p-value**

SBP (mmHg)	140.9 ± 20.9	137.0 ± 23.4	126.9 ± 17.0	0.084	121.9 ± 19.8	120.3 ± 19.1	116.7 ± 14.3	0.807
DBP (mmHg)	83.5 ± 11.5	83.7 ± 11.0	80.8 ± 11.0	0.632	73.8 ± 10.9	73.9 ± 8.8	70.4 ± 7.1	0.671
WC (cm)	97.9 ± 11.3	99.5 ± 14.3	97.5 ± 11.9	0.842	86.0 ± 8.8	87.0 ± 9.2	96.7 ± 9.3	**0.023**
Glyc (mg/dL)	98.3 ± 12.8	110.7 ± 43.5	104.1 ± 37.7	0.332	88.0 ± 9.1	87.4 ± 9.2	87.6 ± 4.5	0.968
HDL-C (mg/dL)	36.6 ± 6.3	36.5 ± 7.4	39.2 ± 8.0	0.371	44.4 ± 7.9	46.7 ± 9.9	42.5 ± 9.5	0.423
TG (mg/dL)	177.2 ± 72.6	172.8 ± 77.2	199.2 ± 109.6	0.542	99.2 ± 27.6	116.5 ± 39.1	132.2 ± 52.4	0.058

**rs699**	**TT (n = 11)**	**TC (n = 43)**	**CC (n = 29)**	**p-value**	**TT (n = 9)**	**TC (n = 32)**	**CC (n = 26)**	**p-value**

SBP (mmHg)	140.5 ± 25.3	136.7 ± 24.8	134.3 ± 14.4	0.726	121.7 ± 16.6	121.9 ± 17.3	117.6 ± 21.8	0.677
DBP (mmHg)	87.4 ± 13.1	82.5 ± 12.4	82.2 ± 8.0	0.385	72.4 ± 13.6	73.5 ± 8.4	72.6 ± 8.4	0.905
WC (cm)	103.7 ± 13.4	99.2 ± 10.9	95.6 ± 14.1	0.164	87.8 ± 14.9	87.7 ± 8.3	86.7 ± 8.9	0.917
Glyc (mg/dL)	94.5 ± 11.6	104.8 ± 28.0	107.8 ± 44.9	0.535	90.0 ± 6.2	86.8 ± 8.8	88.4 ± 9.4	0.586
HDL-C (mg/dL)	42.3 ± 7.3	36.0 ± 6.2	36.8 ± 7.7	**0.031**	46.1 ± 7.4	45.9 ± 9.6	44.5 ± 9.7	0.829
TG (mg/dL)	170.0 ± 72.1	183.7 ± 76.1	178.9 ± 98.2	0.885	114.9 ± 27.2	105.6 ± 29.9	118.1 ± 47.8	0.443

**rs5186**	**AA (n = 45)**	**AC (n = 33)**	**CC (n = 5)**	**p-value**	**AA (n = 37)**	**AC (n = 28)**	**CC (n = 3)**	**p-value**

SBP (mmHg)	134.3 ±18.5	138.4 ± 25.1	141.4 ±25.6	0.624	120.5 ± 19.8	121.9 ± 18.2	105.8 ± 7.7	0.378
DBP (mmHg)	82.9 ± 9.9	83.6 ± 12.8	79.5 ± 11.4	0.746	72.5 ± 10.2	75.3 ± 8.2	65.2 ± 5.1	0.148
WC (cm)	98.8 ± 11.3	99.6 ±14.1	88.4 ± 9.2	0.170	87.4 ± 9.7	86.6 ± 8.9	97.6 ± 9.4	0.160
Glyc (mg/dL)	106.5 ± 33.7	103.0 ± 35.8	95.9 ± 13.4	0.761	86.2 ± 10.5	89.5 ± 6.0	89.6 ± 4.1	0.307
HDL-c (mg/dL)	36.4 ± 6.8	37.8 ± 8.0	38.5 ± 3.1	0.643	46.5 ± 8.5	44.3 ± 9.9	42.3 ± 12.5	0.536
TG (mg/dL)	185.5 ± 95.3	177.5 ± 67.2	150.4 ± 64.4	0.656	104.9 ± 31.8	118.3 ± 36.9	141.9 ± 86.2	0.133

MetS: metabolic syndrome; No MetS: no metabolic syndrome; SBP: systolic blood pressure; DBP: diastolic blood pressure; WC: waist circumference; Glyc: glycaemia; HDL-c: high density lipoprotein-cholesterol; TG: triglycerides.

Considering the differences observed by gender in genotype distribution, a genotype influence analysis on the components of MetS by gender for all variables was developed (data not shown). In this analysis, we observed that the difference of waist circumference for DD genotype is present only in women without MetS (p = 0.005). On the other side, the HDL-cholesterol comparison in men with MetS carriers of the TT genotype *versus* the combination of TC and CC of rs699 (additive model), shows that the latter have lower concentrations of HDL-cholesterol (TT: 40.7 ± 5.2 mg/dL *vs*. TC+CC: 33.2 ± 0.9; p = 0.022), on the contrary, women did not show this difference (p = 0.07).

## DISCUSSION

Conventionally, RAS polymorphisms have been associated to hypertension, a key MetS component. As described, the results of our study suggest RAS polymorphisms are associated with MetS and particularly to the development of some of its components; this is evidenced by the differences in waist circumference and HDL-cholesterol concentration. However, there is great variability in the results obtained by different researchers, which can be attributed to the ethnic and/or sociocultural origins of the populations studied, as for the characteristics of the study design (9,11-13,24).

Insulin resistance is a key component of metabolic syndrome, hypertension and type 2 diabetes mellitus, which is related to RAS performance. This link is evident from the favorable effect of different RAS pharmacological interventions with positive effects on this condition. The first observations related to this fact come from several clinical trials of the late 90s and 2000, in which it was observed that inhibition of the renin-angiotensin system using ACE inhibitors (ACEi) or angiotensin receptor 1 blockers blockers (ARB) had the ability to reduce the establishment of DM2 in individuals at high risk (25,26). After this, many studies have supported the benefits of RAS inhibition in the context of diabetes and cardiovascular disease. For instance, Zreikat and cols. in a cohort of 777 hypertensive patients with MetS observed that ACEi or ARB users would have a decreased risk of cardiovascular events, particularly coronary events (27).

The mechanisms underlying this effect are diverse and complex due to RAS components are produced locally by multiple tissues and not only by the classical pathway system (3). In this context, the RAS plays a key role in the development of associated MetS alterations, since it is capable of inducing hypertension through its vasoconstrictor and sodium retention effect, adding an over activation effect of the Sympathetic Nervous System (SNS) in MetS individuals (28), favoring the development of obesity by affecting satiety, energy expenditure, and growth and differentiation of adipocytes (29), pancreatic islet damage, particularly in the beta cells of the pancreas by reducing their proliferation and stimulate apoptosis (30), and decreasing the insulin secretion and sensitivity (31). In the context of cellular metabolism, three mechanisms by which the RAS, would be altering glucose metabolism and promoting insulin resistance and consequently the development of MetS are described: (a) inhibition of the activation of the insulin receptor substrate-1 (IRS-1)/phosphatidylinositol 3-kinase (PI3K) pathway; (b) the inhibition of differentiation of preadipocytes, responsible for the production of inflammatory or diabetogenic cytokines; and (c) increase in ROS production, damaging pancreatic B cells and causing endothelial dysfunction (32).

Because of the intricate and multifactorial characteristics underlying the development of MetS, is difficult to estimate the implications of genetic variants studied on the pathogenesis of MetS and its components from a viewpoint of associated biological mechanisms and not just in the context of the association. In particular, rs4340 polymorphism has been associated with higher levels of the enzyme in DD genotype when compared to the ID genotype, intermediate level, and genotype II, low level, thus showing its influence on the activity of the system based on the production of greater amounts of angiotensin II (33); rs699 would be associated with increased gene expression of the angiotensinogen gene, however, since the polymorphism causes a substitution of methionine for threonine at exon 2, its effect would not be exerted directly, but it would be a consequence of the unbalance linkage disequilibrium related to this variant, with others located in the promoter region of the gene (34); meanwhile, the rs5186 variant affects the level of AGTR-1 expression, due to the presence of the A allele allows miR-155 to downregulate the expression of the receptor, but not the C allele, which affects miR-155 complementarity, increasing its expression (35). Bearing in mind this background, the analysis of the influence of these polymorphisms becomes more complex due to the difficulty of quantifying the magnitude of their impact on the system activity, for example, in 2002 the functional effect of genetic variants of AGT on its plasma levels was evaluated in healthy individuals, determining that none of the genotypes generated more than 5% variation, in particular rs699 -4.1% only in adults (± 41 years) and not in young (± 15) (36). Thus, given the biological effects of these variants, it would be expected that the combination of risk genotypes, in this case DD of *ACE*, CC of rs699 and CC of *AGTR-1*, confer greater likelihood of developing MetS, DM2, hypertension and cardiovascular disease. However, from our perspective it becomes clear that this is a difficult issue to quantify accurately due to gene-gene and gene-environment underlying interactions, contributing to variability of the results observed in association studies.

Another interesting aspect of our results is related to the observed differences in the magnitudes of the components of MetS, such as the genotypic and allelic frequencies, which were only evidenced when analyzing the variables by gender. This situation is not new, since in recent years gender-dependent associations have been described and also differences in the concentrations and activity levels in the different components of RAS. For example Reyes-Engel indicated that the DD genotype influences higher levels of Ang I in women *versus* men (37), while Bouwman and cols. described an increased risk of weight gain in a period of 10 years for genotype II male carriers, which also contrasts the evidence supporting the role of increased risk associated with the D allele (38). In the same way, hypertension, dyslipidemia and nonalcoholic fatty liver disease (NAFLD), conditions related to MetS, also show a differential manifestation associated to gender (39-41).

An important limitation for the results obtained in the present work is the restricted sample size. Hence, this study should be interpreted in the context of its design since the low number of patients enrolled may be introducing bias to the associations observed. Therefore, future studies encompassing a wider cohort are needed to corroborate, or rule out, the results obtained from this investigation.

In conclusion, the intricate interactions of RAS complicate to understand completely the biological mechanisms that underlie the associations described by various authors; however, our research identified that genetic variants studied actually have an influence on organic metabolism, which also influences the anthropometric and biochemical characteristics of the studied population and would be predisposing factors to cardiometabolic disorders with a strong gender component in the observed associations.
